# Allogeneic stem cell transplant in patients with acute myeloid leukemia and karnofsky performance status score less than or equal to 80%: A study from the acute leukemia working party of the European Society for Blood and Marrow Transplantation (EBMT)

**DOI:** 10.1002/cam4.3593

**Published:** 2020-11-26

**Authors:** Francesco Saraceni, Myriam Labopin, Edouard Forcade, Nicolaus Kröger, Gerard Socié, Riitta Niittyvuopio, Jan J. Cornelissen, Hélène Labussière‐Wallet, Didier Blaise, Goda Choi, Jenny L. Byrne, Gaelle Guillerm, Tony Marchand, Jordi Esteve, Ali Bazarbachi, Bipin Savani, Attilio Olivieri, Arnon Nagler, Mohamad Mohty

**Affiliations:** ^1^ Hematology and Stem Cell Transplant Università Politecnica delle Marche Ancona Italy; ^2^ EBMT Paris Study Office Saint Antoine Hospital Paris France; ^3^ CHU Bordeaux Hôpital Haut‐leveque Pessac France; ^4^ Bone Marrow Transplantation Centre University Hospital Eppendorf Hamburg Germany; ^5^ Department of Hematology ‐ BMT Hopital St. Louis Paris France; ^6^ HUCH Comprehensive Cancer Center Stem Cell Transplantation Unit Helsinki Finland; ^7^ Department of Hematology Erasmus MC Cancer Institute University Medical Center Rotterdam Rotterdam The Netherlands; ^8^ Centre Hospitalier Lyon Sud Lyon France; ^9^ Programme de Transplantation & Therapie Cellulaire Centre de Recherche en Cancérologie de Marseille Institut Paoli Calmettes Marseille France; ^10^ University Medical Center Groningen (UMCG) Department of Hematology University of Groningen Groningen The Netherlands; ^11^ Nottingham University Nottingham United Kingdom; ^12^ C.H.R.U de Brest Service Onco‐Hematologie Brest France; ^13^ Service d`Hematologie Clinique Adulte Centre Hospitalier Universitaire de Rennes Rennes France; ^14^ Hospital Clínic Barcelona Spain; ^15^ Bone Marrow Transplantation Program Department of Internal Medicine American University of Beirut Beirut Lebanon; ^16^ Vanderbilt University Medical Center Nashville TN USA; ^17^ Department of Bone Marrow Transplantation Chaim Sheba Medical Center Tel‐Hashomer Israel

**Keywords:** acute myeloid leukemia, allogeneic stem cell transplant, karnofsky performance status score, myeloablative conditioning, reduced intensity conditioning

## Abstract

Limited data are currently available on the outcome of patients with acute myeloid leukemia (AML) undergoing allogeneic stem cell transplantation (allo‐SCT) with a reduced performance status. We herein present the results of a registry study on 2,936 AML patients undergoing allo‐SCT in first remission (CR1) with a Karnofsky Performance Status (KPS) score less than or equal to 80%. Two‐year leukemia‐free survival (LFS), overall survival (OS) and graft‐versus‐host disease (GVHD)‐free, and relapse‐free survival (GRFS) rates were 54%, 59%, and 41%, respectively. In multivariable analysis, patients with a KPS score = 80% had lower non‐relapse mortality (NRM) and superior OS in comparison to patients with a KPS score <80% (*p* < 0.001). In the subgroup of patients with a KPS score =80%, a reduced‐intensity conditioning (RIC) regimen was associated with an increased risk of relapse (*p* = 0.002) and lower GRFS (*p* < 0.001) compared to myeloablative conditioning (MAC). Differently, in patients with a KPS score <80%, a RIC regimen resulted in lower NRM (*p* < 0.001), whereas relapse incidence did not differ, thus leading to an improved GRFS (*p* = 0.008) as compared to MAC. A transplant from a matched sibling donor (MSD) was associated with a reduced incidence of grade III‐IV acute GVHD (*p* < 0.01) and NRM (*p* < 0.01) in comparison to other donor types. In conclusion, allo‐SCT appears feasible in AML patients with a jeopardized KPS score. Survival is significantly affected by the conditioning intensity, which should be adjusted according to the severity of KPS impairment.

## Graphical abstract

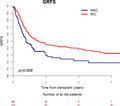

## 1 BACKGROUND

Allogeneic stem cell transplant (allo‐SCT) is a mainstay of post‐remission treatment for acute myeloid leukemia (AML). Nevertheless, this procedure is saddled with a significant risk of mortality, especially in patients undergoing transplant with an impaired physical condition. Different models have been designed with the aim to identify the patients that are able to tolerate a transplant, and to adjust the procedure according to patient fitness. Commonly used scales are the Charlson Comorbidity Index (CCI),^1^ Hematopoietic Cell Transplantation‐specific Comorbidity Index (HCT‐CI),^2^ and Karnofsky Performance Status (KPS) score,^3^ each catching different aspects of patient condition before transplant. KPS represents a robust measure of global health status, and a reliable predictor of non‐relapse mortality and survival after transplant for different hematological malignancies.^4^, ^5^, ^6^, ^7^, ^8^, ^9^ Furthermore, it has the advantage of allowing a dynamic assessment which is extremely easy to perform. Historical data show a dismal outcome for patients undergoing transplant with a jeopardized performance score.^10^, ^11^, ^12^, ^13^, ^14^ However, with the availability of low‐intensity induction regimens for AML,^15^, ^16^, ^17^ the recent design of different reduced‐intensity conditioning (RIC) protocols,^18^, ^19^, ^20^ the expansion of donor sources,^21^ and the significant improvement in supportive care,^22^, ^23^ an increasing number of AML patients with a jeopardized performance score are considered for an allo‐SCT. Nevertheless, limited evidence is available about transplant outcomes in this delicate setting. We herein present the results of a retrospective analysis conducted on a large, homogeneous cohort of AML patients with a KPS score less than or equal to 80%, undergoing allogeneic transplant in first remission (CR1).

## 2 PATIENTS AND METHODS

We included in the analysis patients diagnosed with AML older than 18 years, who underwent an allo‐SCT in first complete remission between 2000 and 2018, with a KPS score at the time of transplant ranging from 50% to 80%. Patients received a transplant from a matched sibling donor (MSD), matched unrelated donor (MUD), mismatched unrelated donor at one HLA locus (MMUD), cord blood (CB), or haploidentical (haplo) donor. All transplants from haplo donors were performed using an unmanipulated, T‐cell replete graft. Conditioning intensity (myeloablative, MAC; reduced intensity, RIC) was defined according to EBMT standards.^24^ A complete list of EBMT centers contributing data to this study is presented in Table S4, Supplementary file.

Non‐relapse mortality (NRM) was defined as death without prior recurrence of the disease. Relapse was defined according to standard criteria for AML. Leukemia‐free survival (LFS) was defined as survival in the absence of relapse. Overall survival (OS) was estimated from the day of transplant until death or last follow‐up. Graft‐versus‐host disease (GVHD)‐free, relapse‐free survival (GRFS) was defined by the first of the following events: acute grade III‐IV GVHD, severe chronic GVHD, relapse, or death. LFS, OS, and GRFS were calculated using the Kaplan–Meier method; NRM, relapse, and GVHD were calculated by cumulative incidence analysis considering competing risks. For univariate comparisons, the log‐rank test was employed for LFS, OS, and GRFS, whereas the Gray's test was used for GVHD, relapse incidence, and NRM. The Cox model was employed for multivariate analyses. In order to investigate prognostic factors in this population, all factors associated with one outcome in univariate analysis with a p value less than 0.05 or variables deemed conceptually important were included in the Cox model. We investigated more specifically conditioning intensity in the population of patients receiving transplant from MSD or MUD. As we found a qualitative interaction between conditioning intensity and KPS, further analyses were stratified on KPS value equal or less than 80. All p‐values were two‐sided, and *p* < 0.05 was considered statistically significant. Statistical analyses were performed with the SPSS 24 (SPSS Inc./IBM, Armonk, NY, USA) and R 3.6.2 (R Development Core Team, Vienna, Austria) software packages.

## 3 RESULTS

### 3.1 Patient, disease, and transplant characteristics

A total of 2,963 patients were included in the study. The median age at transplant was 55 years (range 18‐77 years). Patients with secondary AML were older as compared to patients with de novo AML. The KPS score was =80% in 85% of the patients and <80% in 15% of the patients. Donor type was MSD, MUD, MMUD, haplo, or CB in 47%, 35%, 8%, 6%, and 4% of the patients, respectively. A myeloablative (MAC) or reduced‐intensity (RIC) conditioning was administered in 42% and 58% of the patients, respectively. Most common MAC regimens included busulfan and cyclophosphamide (BuCy, 28%), busulfan and fludarabine (BuFlu with busulfan dose equal to or greater than 9.6 mg/kg, 27%), or cyclophosphamide and total‐body irradiation (CyTBI, 23%); most common RIC regimes included BuFlu (busulfan dose equal to or lower than 6.4 mg/kg, 47%), fludarabine and melphalan (FluMel, 11%), and fludarabine‐TBI (16%). Conditioning regimens details are presented in the supplementary file, Table S1.

Graft‐*versus*‐host disease (GVHD) prophylaxis regimens included cyclosporine combined with methotrexate (CSA+MTX) or with mycophenolate mofetil (CSA+MMF) or CSA alone in 37%, 27%, and 15% of the patients, respectively. Anti‐thymocyte globulin (ATG) was administered to 55% of the patients. Patient characteristics are detailed in Table 1.

**Table 1:** Patient characteristics

	KPS score =80%	KPS score <80%	*p* value
Total number included in analysis	2522	441	
Follow up (months), median (IQR)	35.4 (12.3 ‐ 89.7)	25.3 (12.3 ‐ 61.9)	0.001
Age of patient at HSCT (years), Median (range) (IQR)	55 (18 ‐ 76) (44 – 62)	56 (19‐77) (48‐62)	0.05
Year of transplant, median (range)	2014 (2000‐2018)	2015 (2000‐2018)	0.19
Diagnosis, n (%)			
De novo AML	2146 (85%)	348 (79%)	0.001
Secondary AML	376 (15%)	93 (21%)	
Gender of patient, n (%)			
Male	1311 (52%)	211 (48%)	0.1
Female	1208 (48%)	230 (52%)	
HCT‐CI at transplant, n (%)			
HCT‐CI =0	667 (52%)	108 (41%)	0.01
HCT‐CI 1‐2	276 (21%)	69 (26%)	
HCT‐CI >2	347 (27%)	84 (32%)	
Missing	1232	180	
Interval from diagnosis to SCT (months), median (range) (IQR)	4.7 (0.7‐17.8) (3.8‐6.1)	4.9 (0.8‐17.9) (3.8‐6.3)	0.22
Interval from diagnosis to CR1 (days), median (range) (IQR)	49 (1‐476) (36‐73)	52 (20‐401) (37‐78)	0.2
Interval from CR1 to SCT (days), median (range) (IQR)	91 (2‐391) (59‐126)	90 (10‐341) (57‐140)	0.55
Cytogenetics, n (%)			
Favorable	155 (6%)	17 (4%)	0.12
Intermediate	1712 (68%)	299 (68%)	
Adverse	655 (26%)	125 (28%)	
WBC at diagnosis (x 10^9^/L), median (range) (IQR)	7.8 (0.2‐665) (2.4‐40)	7.0 (0.3‐790) (2.5‐37.9)	0.4
Donor Type, n (%)			
MSD	1186 (47%)	221 (50%)	0.18
UD 10/10	889 (35%)	139 (32%)	
UD 9/10	206 (8%)	34 (8%)	
TR Haplo	161 (6%)	25 (6%)	
CBT	80 (3%)	22 (5%)	
Donor/recipient sex mismatch, n (%)			
F‐>M	2078 (83%)	366 (83%)	0.86
No F‐>M	436 (17%)	75 (17%)	
Stem cell source, n (%)			
BM	367 (15%)	40 (9%)	0.002
PBSCs	2075 (82%)	379 (86%)	
CB	80 (3%)	22 (5%)	
Conditioning intensity, n (%)			
MAC	1108 (44%)	144 (33%)	<0.001
RIC	1414 (56%)	297 (67%)	
ATG used, n (%)			
No	1145 (45%)	176 (40%)	0.032
Yes	1377 (55%)	265 (60%)	

Some percentages do not add up to 100% because of rounding.

Abbreviations: AML, acute myeloid leukemia; ATG, anti‐thymocyte globulin; BM, bone marrow; CB; cord blood; GVHD, graft‐versus‐host disease; KPS, Karnofsky performance status; LFS, leukemia‐free survival; MAC, myeloablative conditioning; MSD, matched sibling donor; NRM, non‐relapse mortality; OS, overall survival; PBSCs, peripheral blood stem cells; RI, relapse incidence; RIC, reduced‐intensity conditioning; UD, unrelated donor; WBC, white blood cells.

### 3.2 Engraftment and graft‐versus‐host disease

Successful engraftment was achieved by 98% of the patients. The median day of neutrophil recovery (defined as achieving an absolute neutrophil count of 500/L) was 18 (range 2‐72). Cumulative incidence of grade II‐IV and III‐IV aGVHD was 26% and 8%, respectively. Cumulative incidence of cGVHD at 2 years was 38%; severe cGVHD was observed in 18% of the patients. Incidence of grade II – IV aGVHD was lower after MSD transplant as compared to other donor types (MUD: *p* = 0.001; MMUD: *p* < 0.001; haplo: *p* < 0.001; CB: *p* < 0.001; MSD as reference, Table 2). Similarly, a transplant from a female donor was independently associated with an increased rate of grade III‐IV aGVHD (*p* = 0.03). Furthermore, donor type was independently associated with the incidence of severe cGVHD: compared to the reference group MSD, CB transplant resulted in inferior incidence (*p* = 0.009), whereas 10/10 UD resulted in higher incidence (*p* = 0.007) of severe cGVHD, respectively. A non‐significant trend toward a reduced incidence of severe cGVHD (*p* = 0.06) was noted for haploidentical transplantation as compared to other donor types. Notably, ATG administration resulted in a lower incidence of grade III‐IV aGVHD (*p* < 0.001) and severe cGVHD (*p* < 0.001).

**Table 2:** Multivariate analysis of transplant outcomes

Outcome		HR (95% CI)	*p*
RI	Secondary AML vs de novo AML	1.3 (1.02‐1.5)	0.03
	Good risk (reference)	1	
	Intermediate risk	1.2 (0.8‐1.8)	0.4
	Poor risk	2.2 (1.45‐3.31)	<0.001
	MSD (reference)	1	
	UD 10/10	0.8 (0.698‐1.02)	0.09
	UD 9/10	1.1 (0.854‐1.52)	0.4
	Haplo	0.9 (0.602‐1.25)	0.4
	CB	0.7 (0.436‐1.14)	0.2
NRM	Age (per 10 years)	1.3 (1.17‐1.41)	<0.001
	Secondary AML vs de novo AML	1.4 (1.13‐1.77)	0.002
	KPS =80% vs <80%	0.6 (0.479‐0.752)	<0.001
	MSD (reference)		
	UD 10/10	1.4 (1.1‐1.79)	0.006
	UD 9/10	2.4 (1.75‐3.28)	<0.001
	Haplo	1.8 (1.2‐2.57)	0.004
	CB	2.04 (1.32‐3.16)	0.001
	Female vs male patient	0.8 (0.647‐0.937)	0.008
	Patient CMV positive serology	1.3 (1.01‐1.55)	0.04
LFS	Age (per 10 years)	1.1 (1.03‐1.16)	0.002
	Secondary AML vs de novo AML	1.3 (1.14‐1.53)	<0.001
	Good risk (reference)		
	Intermediate risk	1.2 (0.857‐1.56)	0.3
	Poor risk	1.8 (1.31‐2.42)	<0.001
	KPS =80% vs <80%	0.7 (0.636‐0.867)	<0.001
	MSD (reference)		
	UD 10/10	1.03 (0.888‐1.2)	0.7
	UD 9/10	1.6 (1.26‐1.92)	<0.001
	Haplo	1.2 (0.909‐1.53)	0.2
	CB	1. (0.831‐1.58)	0.4
OS	Age (per 10 years)	1.2 (1.1‐1.24)	<0.001
	Secondary AML vs de novo AML	1.3 (1.12‐1.54)	<0.001
	Good risk (reference)		
	Intermediate risk	1.1 (0.815‐1.57)	0.5
	Poor risk	1.7 (1.21‐2.36)	0.002
	KPS =80% vs <80%	0.7 (0.564‐0.779)	<0.001
	MSD (reference)		
	UD 10/10	1.1 (0.92‐1.27)	0.3
	UD 9/10	1.7 (1.34‐2.1)	<0.001
	Haplo	1.2 (0.891‐1.58)	0.2
	CB	1.2 (0.848‐1.68)	0.3
	Female vs male patient	0.9 (0.752‐0.973)	0.02
	Patient CMV positive serology	1.2 (0.997‐1.34)	0.06
GRFS	Secondary AML vs de novo AML	1.2 (1.05‐1.39)	0.007
	Good risk (reference)		
	Intermediate risk	1.3 (0.986‐1.67)	0.06
	Poor risk	1.8 (1.36‐2.36)	<0.001
	KPS =80% vs <80%	0.8 (0.712‐0.947)	0.006
	MSD (reference)		
	UD 10/10	1.2 (1.01‐1.32)	0.03
	UD 9/10	1.5 (1.26‐1.86)	<0.001
	Female vs male patient	0.85 (0.762‐0.94)	0.002
	Female vs male donor	1.2 (1.04‐1.29)	0.006
	ATG used	0.8 (0.733‐0.926)	0.001
aGVHD III‐IV	Good risk (reference)		
	Intermediate risk	2.5 (1.11‐5.73)	0.03
	Poor risk	2.3 (0.988‐5.35)	0.05
	MSD (reference)		
	UD 10/10	1.8 (1.28‐2.63)	0.001
	UD 9/10	3 (1.85‐4.77)	<0.001
	Haplo	2.6 (1.58‐4.32)	<0.001
	CB	3.7 (2.17‐6.36)	<0.001
	Female vs male donor	1.4 (1.04‐1.78)	0.03
	ATG used	0.6 (0.441‐0.802)	<0.001
	Patient CMV positive serology	0.7 (0.541‐0.957)	0.02
cGVHD	KPS =80% vs <80%	0.8 (0.667‐0.988)	0.04
	MSD (reference)		
	UD 10/10	1.2 (1.03‐1.46)	0.02
	UD 9/10	1.4 (1.02‐1.79)	0.04
	Haplo	0.7 (0.523‐1.06)	0.09
	CB	0.5 (0.329‐0.818)	0.004
	Female vs male patient	0.8 (0.741‐0.973)	0.02
	Female vs male donor	1.3 (1.15‐1.52)	<0.001
	ATG used	0.6 (0.552‐0.75)	<0.001
Severe cGVHD	MSD (reference)		
	UD 10/10	1.4 (1.1‐1.83)	0.007
	UD 9/10	1.2 (0.755‐1.83)	0.5
	Haplo	0.6 (0.331‐1.02)	0.06
	CB	0.3 (0.148‐0.763)	0.009
	Female vs male patient	0.7 (0.6‐0.895)	0.002
	Female vs male donor	1.6 (1.28‐1.92)	<0.001
	ATG used	0.5 (0.427‐0.672)	<0.001

Note:

Only variables with *p* values <0.1 were included.

Abbreviations: ATG, anti‐thymocyte globulin; CB, cord blood transplant; CMV, Cytomegalovirus; GRFS, graft‐versus‐host disease‐free, relapse‐free survival; GVHD, graft‐versus‐host disease; Haplo, haploidentical transplant; KPS, Karnofsky performance status; LFS, leukemia‐free survival; MSD, matched sibling donor; NRM, non‐relapse mortality; OS, overall survival; RI, relapse incidence; UD, unrelated donor.

### 3.3 Non‐relapse mortality, relapse, and survival

Two‐year NRM and relapse rates were 19% and 27%, respectively. Common causes of death included leukemia relapse (44%), infection (21%), and GVHD (19%).

**Figure 1:**
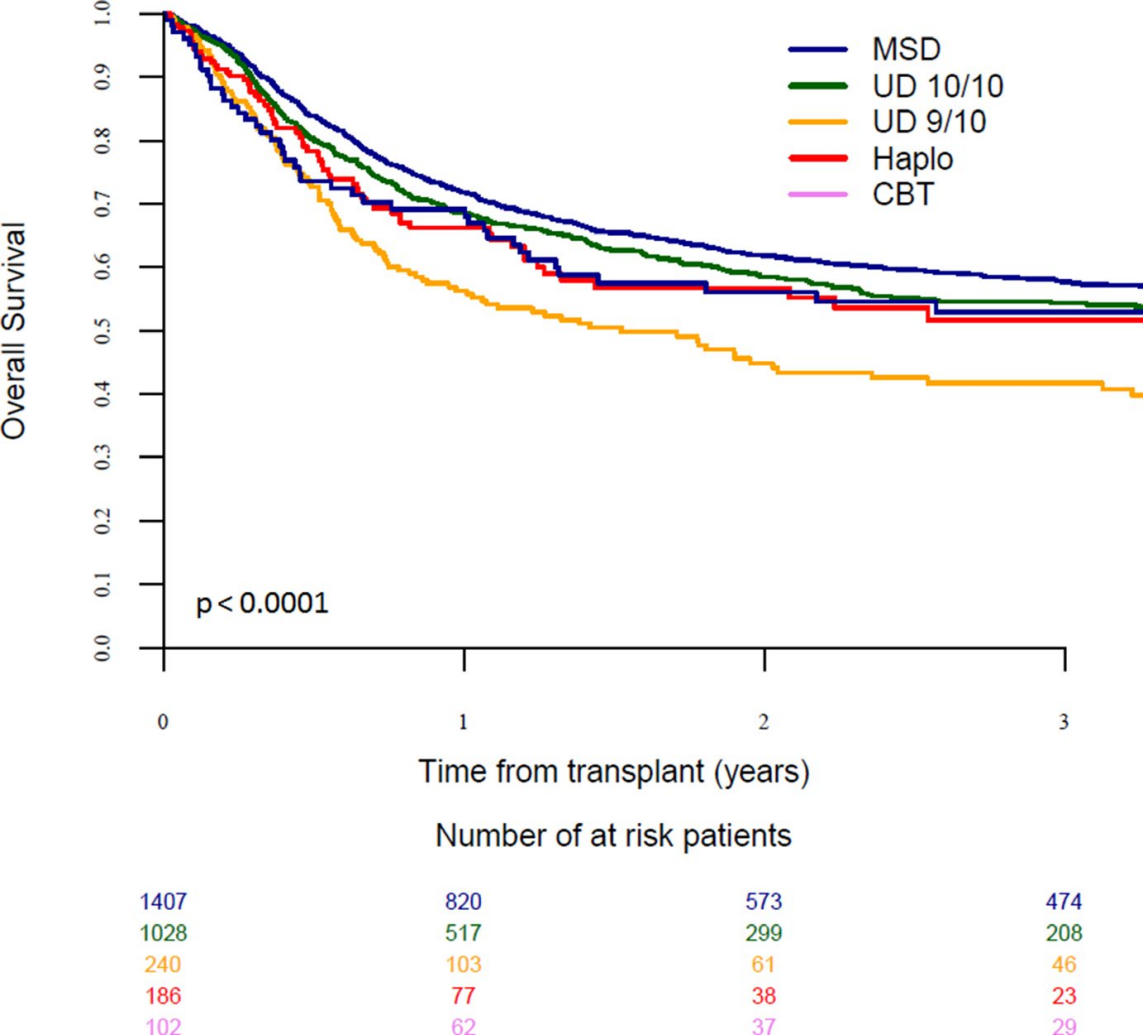
Overall survival in patients with KPS score less than or equal to 80% according to donor type

Leukemia‐free survival (LFS), OS and GRFS rates were 54%, 59%, and 41%, respectively. In multivariable analysis, MSD (reference group) resulted in a significantly reduced risk of NRM as compared to other donor types (MUD: *p* = 0.006; MMUD: *p* < 0.001; haplo: *p* = 0.004; CB: *p* = 0.001, Table 2). MUD transplant resulted in inferior GRFS (*p* = 0.03), whereas MMUD was associated with inferior LFS, OS, and GRFS (*p* < 0.001) as compared to MSD (Figure 1, Table 2). There was no significant difference in survival outcomes following haplo or CB compared to MSD transplant.

We evaluated the influence of KPS score on NRM, comparing patients with KPS =80% with patients with KPS <80%. Patients with a KPS <80% showed higher non‐relapse mortality and inferior survival when compared to patients with a KPS =80% (NRM: *p* < 0.01; OS: *p* < 0.001). Other factors associated with poor survival in multivariable analysis were older patient age, male gender, poor‐risk cytogenetics, and secondary AML (Table 2). In particular, patients with secondary AML had significantly higher relapse and lower OS as compared to patients with de novo AML (*p* = 0.012, *p* < 0.0001, respectively). In this subgroup, KPS score <80% remained associated with higher NRM and inferior OS as compared to KPS =80% (*p* = 0.003, *p* = 0.001, respectively).

**Figure 2:**
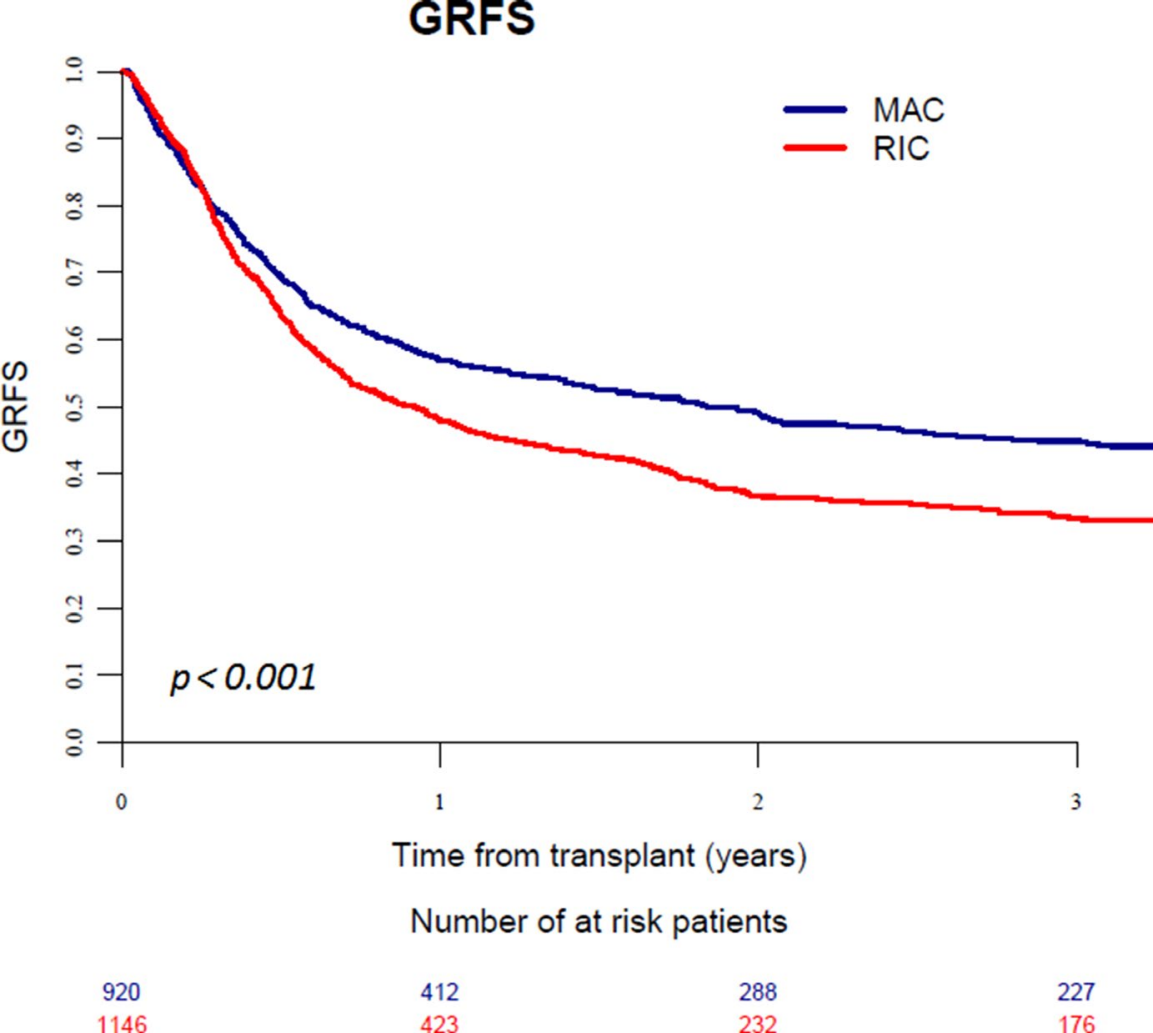
Graft‐versus‐host disease‐free, relapse‐free survival in patients with KPS score =80% receiving MAC or RIC regimen

**Figure 3:**
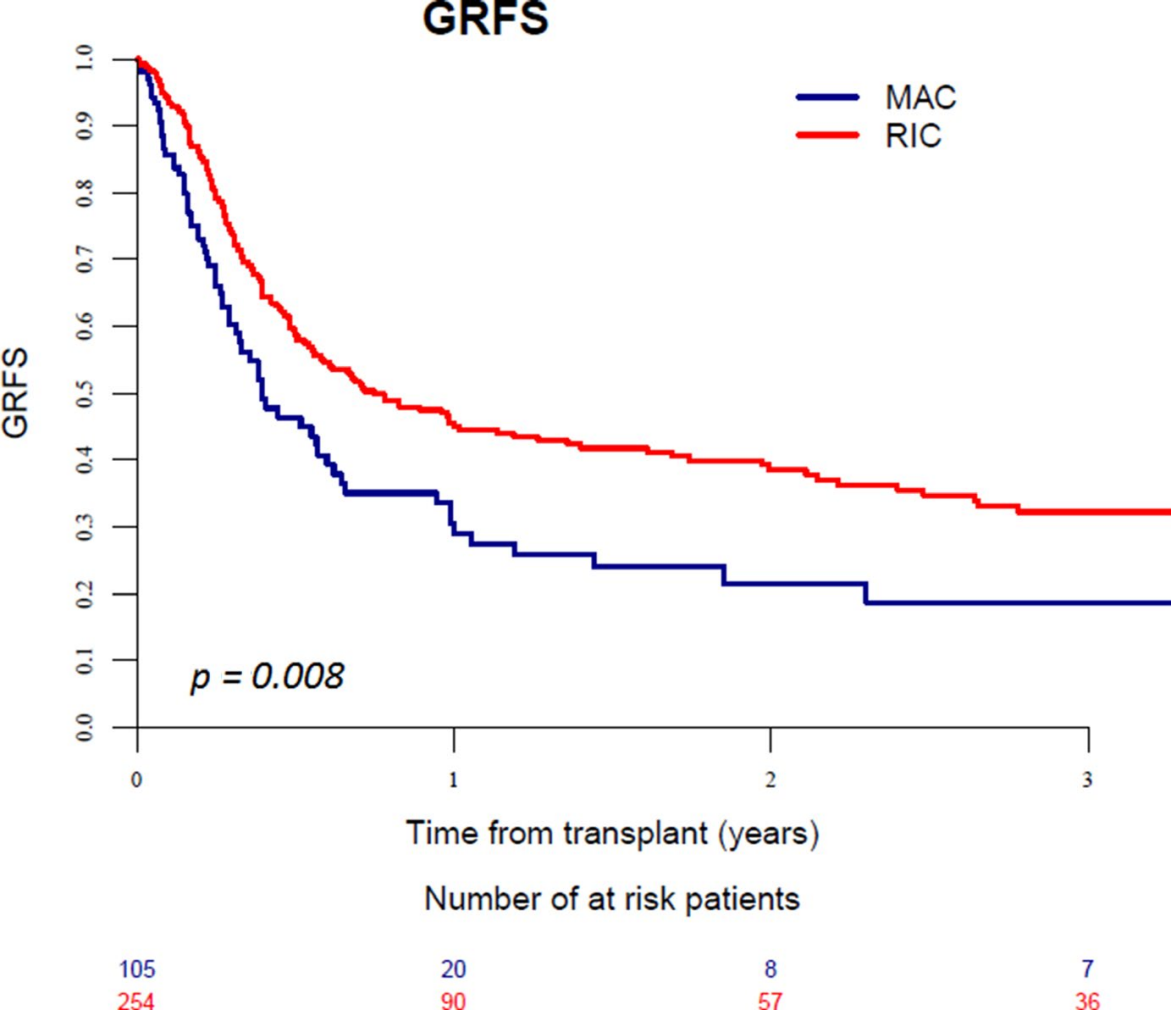
Graft‐versus‐host disease‐free, relapse‐free survival in patients with KPS score <80% receiving MAC or RIC regimen

When comparing MAC and RIC conditioning regimens in patients receiving a MSD or MUD transplant, we found a significant interaction between conditioning intensity and KPS score. Consequently, the two groups of patients according to KPS score (= 80% or <80%) were analyzed separately (Table S2, supplementary file). In the cohort of patients with a KPS score =80%, a RIC regimen was associated with higher relapse (*p* = 0.002), and lower GRFS (*p* < 0.001) in comparison to those seen with MAC (Figure 2), whereas NRM was similar (*p* = 0.2). Differently, in patients with a KPS score <80%, a RIC regimen resulted in lower NRM (*p* < 0.001), better LFS (*p* = 0.003), OS (*p* < 0.001), and GRFS (*p* = 0.008) in comparison to MAC conditioning, whereas incidence of relapse did not differ (*p* = 0.9; Table 2, Figure 3).

In addition, MUD transplant was associated with higher NRM (*p* = 0.002) and a trend for worse GRFS (*p* = 0.05) in comparison to MSD in patients with a KPS score =80%; no statistically significant difference was observed in the subgroup of patients with KPS <80%. In order to analyze the relative impact of KPS score and HCT‐CI on patients outcomes, we performed a subgroup analysis in patients with available data about HCT‐CI score (n = 1551). When HCT‐CI score was included in multivariable analysis, KPS <80% and HCT‐CI >2 resulted independently associated with higher NRM and inferior survival. (Table S3, supplementary file).

## 4 DISCUSSION

With the development of targeted therapies and low‐intensity protocols, many unfit patients diagnosed with AML are currently offered induction therapy with curative intent and if a remission is obtained, they are considered for an allo‐SCT. This study aimed to analyze the outcome of a homogeneous population of frail AML patients undergoing allo‐SCT in CR1. In our series of patients with a performance status ≤80% we observed encouraging outcomes, with a long‐term survival approaching 60%.

Assessment of patient capability to tolerate the transplant procedure is a major turning point along the AML treatment path. The KPS scale is widely used by clinicians as it represents a robust surrogate of patient global health status, and has been shown to be strongly associated with NRM and survival after transplant.^3^, ^4^, ^5^, ^6^ Interestingly, in a recent study in which the authors aimed to develop a “machine‐learning” based prognostic model for patients with acute leukemia undergoing transplant,^25^ the algorithm included KPS along with the main parameters associated with patient survival. Furthermore, in a recently published analysis by Carrè et al.^25^ KPS emerged as most reliable predictor of NRM when compared to other parameters such as age and HCT‐CI. In our series, a threshold of KPS score set at 80% strongly discriminated patient outcome. In fact, in patients with a KPS score =80%, the NRM rate was remarkably low, resulting in a long‐term survival of above 60%, thus being comparable to the figures expected for fit patients undergoing allo‐SCT.^26^ This finding is in accordance with previous literature, as these patients have a slightly impaired performance status and, if translated to ECOG score, would be included in grade 1.^27^ On the contrary, patients with a KPS score <80% showed high NRM and poor survival. Interestingly, in the subgroup of patients with a KPS =80%, a RIC regimen was associated with an increased incidence of relapse as compared to MAC, whereas NRM did not differ, thus translating into superior survival for MAC as compared to RIC conditioning. In contrast, in the subgroup of patients with a KPS <80%, a RIC regimen resulted in significantly reduced NRM and superior survival in comparison to MAC, in agreement with previous evidence.^6^, ^8^

The choice of the conditioning intensity represents a major conundrum when approaching a patient with an impaired performance status. The recent development of reduced‐intensity regimens has allowed a significant proportion of unfit patients to undergo allo‐SCT; nevertheless, an increased rate of relapse has been reported following RIC as compared to standard myeloablative regimens, especially in patients undergoing transplant with persistence of residual disease.^28^, ^29^ Recent evidence^30^ consolidated the concept that a MAC regimen should not be withheld if the patient is deemed to be capable of tolerating it, and reduced intensity regimens should be reserved for unfit patients. In historical studies, a KPS score ≤80% has been reported to be associated with decreased survival following allo‐SCT,^5^, ^30^, ^31^ thus discouraging clinicians to offer this potentially curative treatment to these patients. Of note, most previous studies included patients with mixed hematological malignancies and with active disease at the time of transplant. In fact, in order to limit confounders, we selected only AML patients undergoing allo‐SCT in CR1. Furthermore, conditioning regimens, donor sources, and supportive therapy have significantly developed recently, leading to a profound evolution in transplant practice.^32^ Indeed, a significant proportion of the patients included in our study received modern myeloablative regimens as fludarabine combined with an alkylator at myeloablative dosage, commonly referred to as “reduced toxicity regimens”. Such protocols are believed to be associated with similar anti‐leukemic effect but better tolerability as compared to standard protocols as busulfan and cyclophosphamide or cyclophosphamide and total body irradiation,^19^ and should be regarded as protocols with intermediate intensity, sharing both MAC and RIC features, as shown by a recent study.^33^

Globally, the findings of the present analysis emphasize the strong predictive power of KPS score in AML patients undergoing allo‐SCT; furthermore, they shed new light on its impact on clinical practice. First, a reduced performance status should not be considered *per*‐*se* a major obstacle in performing a transplant in a patient with AML in CR1. Moreover, these data suggest to carefully distinguish between patients with a slightly reduced KPS score (i.e., =80%) and those with a markedly impaired performance status (i.e., <80%), when planning a transplant and selecting the conditioning regimen. In fact, a MAC regimen should not be withheld in the former patients, whereas a RIC protocol is associated with superior outcome in the latter category.

Interestingly, when HCT‐CI score was included in multivariable analysis, KPS <80% and HCT‐CI >2 resulted independently associated with higher NRM and inferior survival. This finding is consistent with previous evidence.^4^ In fact, HCT‐CI and KPS capture different aspects of patient health status, and might be employed as complementary measures of patient fitness before transplant. Unfortunately, the amount of missing data did not allow to draw any definite conclusion about the influence of HCT‐CI score on outcome in patients with KPS <80%.

When we analyzed different donor types, a transplant from a MSD was associated with better outcome, resulting in reduced incidence of aGVHD and lower NRM rates in comparison to other donor sources. In particular, an unrelated donor resulted in higher risk of GVHD, whereas MMUD transplant predicted significantly inferior survival. Notably, in our study a significant proportion of patients (288, 10% of the study population) received transplantation from CB or a haplo donor. This represents novel data, as the main prognostic models currently available derive from studies excluding patients receiving transplants from alternative donors.^34^, ^35^ Interestingly, we observed a promising outcome in this subgroup of patients receiving a CB or haplo transplant. Survival was similar to matched unrelated donor and superior to mismatched unrelated donor; furthermore, lower rates of severe cGVHD were observed, consistent with recent publications.^36^

This study carries some limitations, mainly related to its retrospective design and non‐randomized patient allocation to different groups, which could have influenced the results. Nevertheless, a prospective, randomized study enrolling frail AML patients undergoing allo‐SCT is not available and hardly likely to be conducted in the near future; thus, the findings of the present analysis could be of guidance for clinical practice.

In conclusion, allogeneic transplant appears feasible in AML patients with a reduced performance score. Outcome varied significantly depending on conditioning intensity, which should be adjusted according to the severity of KPS impairment. In fact, in patients undergoing transplant with a slightly reduced KPS score (=80%), a MAC regimen provided inferior relapse and better survival, whereas in patients with a markedly impaired performance status (< 80%) a RIC conditioning was associated with better outcome as compared to MAC. Regarding donor selection, MSD transplant resulted in lower rates of aGVHD and NRM in comparison to other donor sources; of note, a CB or haplo transplant were associated with a low incidence of severe cGVHD and survival rates which were similar to those observed following MUD and superior to those following MMUD transplant.

## 5 ETHICAL DECLARATIONS

Data were provided and the study design was approved by the Acute Leukemia Working Party (ALWP) of the European society for Blood and Marrow Transplantation (EBMT), in accordance with the EBMT guidelines for retrospective studies. Since 1990, patients have been able to provide informed consent that authorizes the use of their transplant information for research purposes. The ALWP of the EBMT granted ethical approval for this study.

## CONFLICT OF INTEREST

The authors declare no conflict of interest.

## AUTHOR CONTRIBUTIONS

F.S. A.N. and M.M. designed the study, the synopsis of which was approved by the acute leukemia working party of the EBMT; M.L. performed all the statistical analysis; F.S. wrote the first draft of the manuscript; A.N. and M.M. reviewed the manuscript; all co‐authors contributed data to the EBMT registry, read the manuscript and approved the final version.

## Supporting information

Supplementary Material

## Data Availability

All relevant data are available on request at ALWP‐EBMT registry.

## References

[1] Charlson M , Szatrowski TP , Peterson J , et al. Validation of a combined comorbidity index. J Clin Epidemiol. 1994;47:1245‐1251. 7722560 10.1016/0895-4356(94)90129-5

[2] Sorror ML , Maris MB , Storb R , et al. Hematopoietic cell transplantation (HCT)‐specific comorbidity index: a new tool for risk assessment before allogeneic HCT. Blood. 2005;106:2912‐2919. 15994282 10.1182/blood-2005-05-2004 PMC1895304

[3] Karnofsky DA , Burchenal JH . The clinical evaluation of chemotherapeutic agents in cancer In: Macleod CM, ed. Evaluation of Chemotherapeutic Agents. New York, NY: Columbia University Press; 1949:191.

[4] Sorror M , Storer B , Sandmaier BM , et al. Hematopoietic cell transplantation‐comorbidity index and Karnofsky performance status are independent predictors of morbidity and mortality after allogeneic nonmyeloablative hematopoietic cell transplantation. Cancer. 2008;112:1992‐2001. 18311781 10.1002/cncr.23375

[5] Artz AS , Pollyea DA , Kocherginsky M , et al. Performance status and comorbidity predict transplant‐related mortality after allogeneic hematopoietic cell transplantation. Biol Blood Marrow Transplant. 2006;12:954‐964. 16920562 10.1016/j.bbmt.2006.05.015

[6] Gómez‐Núñez M , Martino R , Caballero MD , et al. Elderly age and prior autologous transplantation have a deleterious effect on survival following allogeneic peripheral blood stem cell transplantation with reduced‐intensity conditioning: results from the Spanish multicenter prospective trial. Bone Marrow Transplant. 2004;33:477‐482. 14730333 10.1038/sj.bmt.1704379

[7] Deschler B , Binek K , Ihorst G , et al. Prognostic factor and quality of life analysis in 160 patients aged >or =60 years with hematologic neoplasias treated with allogeneic hematopoietic cell transplantation. Biol Blood Marrow Transplant. 2010;16:967‐975. 20144720 10.1016/j.bbmt.2010.02.004

[8] Luger SM , Ringdén O , Zhang M‐J , et al. Similar outcomes using myeloablative vs reduced‐intensity allogeneic transplant preparative regimens for AML or MDS. Bone Marrow Transplant. 2012;47:203‐211. 21441963 10.1038/bmt.2011.69 PMC3134582

[9] McClune BL , Weisdorf DJ , Pedersen TL , et al. Effect of age on outcome of reduced‐intensity hematopoietic cell transplantation for older patients with acute myeloid leukemia in first complete remission or with myelodysplastic syndrome. J Clin Oncol. 2010;28:1878‐1887. 20212255 10.1200/JCO.2009.25.4821 PMC2860368

[10] Giralt S , Thall PF , Khouri I , et al. Melphalan and purine analog‐containing preparative regimens: reduced‐intensity conditioning for patients with hematologic malignancies undergoing allogeneic progenitor cell transplantation. Blood. 2001;97:631‐637. 11157478 10.1182/blood.v97.3.631

[11] Wong R , Shahjahan M , Wang X , et al. Prognostic factors for outcomes of patients with refractory or relapsed acute myelogenous leukemia or myelodysplastic syndromes undergoing allogeneic progenitor cell transplantation. Biol Blood Marrow Transplant. 2005;11:108‐114. 15682071 10.1016/j.bbmt.2004.10.008

[12] Saraceni F , Labopin M , Brecht A , et al. Fludarabine‐treosulfan compared to thiotepa‐busulfan‐fludarabine or FLAMSA as conditioning regimen for patients with primary refractory or relapsed acute myeloid leukemia: a study from the Acute Leukemia Working Party of the European Society for Blood and Marrow Transplantation (EBMT). J Hematol Oncol. 2019;25(12):44. 10.1186/s13045-019-0727-4 PMC6482556 31023346

[13] Duval M , Klein JP , He W , et al. Hematopoietic stem‐cell transplantation for acute leukemia in relapse or primary induction failure. J Clin Oncol. 2010;28:3730‐3738. 20625136 10.1200/JCO.2010.28.8852 PMC2917308

[14] Welch JS , Petti AA , Miller CA , et al. TP53 and decitabine in acute myeloid leukemia and myelodysplastic syndromes. N Engl J Med. 2016;375:2023‐2036. 27959731 10.1056/NEJMoa1605949 PMC5217532

[15] Cortes JE , Heidel FH , Hellmann A , et al. Randomized comparison of low dose cytarabine with or without glasdegib in patients with newly diagnosed acute myeloid leukemia or high‐risk myelodysplastic syndrome. Leukemia. 2019;33:379‐389. 30555165 10.1038/s41375-018-0312-9 PMC6365492

[16] DiNardo CD , Pratz K , Pullarkat V , et al. Venetoclax combined with decitabine or azacitidine in treatment‐naive, elderly patients with acute myeloid leukemia. Blood. 2019;133:7‐17. 30361262 10.1182/blood-2018-08-868752 PMC6318429

[17] Shimoni A , Hardan I , Shem‐Tov N , et al. Allogeneic hematopoietic stem‐cell transplantation in AML and MDS using myeloablative versus reduced‐intensity conditioning: the role of dose intensity. Leukemia. 2006;20:322‐328. 16307018 10.1038/sj.leu.2404037

[18] Mohty M , Malard F , Blaise D , et al. Reduced‐toxicity conditioning with fludarabine, once‐daily intravenous busulfan, and antithymocyte globulins prior to allogeneic stem cell transplantation: results of a multicenter prospective phase 2 trial. Cancer. 2015;121:562‐569. 25283774 10.1002/cncr.29087

[19] Rambaldi A , Grassi A , Masciulli A , et al. Busulfan plus cyclophosphamide versus busulfan plus fludarabine as a preparative regimen for allogeneic haemopoietic stem‐cell transplantation in patients with acute myeloid leukaemia: an open‐label, multicentre, randomised, phase 3 trial. Lancet Oncol. 2015;16:1525‐1536. 26429297 10.1016/S1470-2045(15)00200-4

[20] Beelen DW , Trenschel R , Stelljes M , et al. Treosulfan or busulfan plus fludarabine as conditioning treatment before allogeneic haemopoietic stem cell transplantation for older patients with acute myeloid leukaemia or myelodysplastic syndrome (MC‐FludT.14/L): a randomised, non‐inferiority, phase 3 trial. Lancet Haematol. 2020;7:2839. 10.1016/S2352-3026(19)30157-7 31606445

[21] Salas MQ , Law AD , Lam W , et al. Safety and efficacy of haploidentical peripheral blood stem cell transplantation for myeloid malignancies using post‐transplantation cyclophosphamide and anti‐thymocyte globulin as graft‐versus‐host disease prophylaxis. Clin Hematol Int. 2019;2:105‐113. 10.2991/chi.d.190316.003 PMC8432390 34595418

[22] Gooley TA , Chien JW , Pergam SA , et al. Reduced mortality after allogeneic hematopoietic‐cell transplantation. N Engl J Med. 2010;363:2091‐2101. 21105791 10.1056/NEJMoa1004383 PMC3017343

[23] Palomo M , Diaz‐Ricart M , Carreras E . Endothelial dysfunction in hematopoietic cell transplantation. Clin Hematol J. 2019;1:45‐51. 10.2991/chi.d.190317.001 PMC8432381 34595410

[24] Bacigalupo A , Ballen K , Rizzo D , et al. Defining the intensity of conditioning regimens: working definitions. Biol Blood Marrow Transplant. 2009;15:1628‐1633. 19896087 10.1016/j.bbmt.2009.07.004 PMC2861656

[25] Shouval R , Labopin M , Bondi O , et al. Prediction of allogeneic hematopoietic stem‐cell transplantation mortality 100 days after transplantation using a machine learning algorithm: A European Group for Blood and Marrow Transplantation Acute Leukemia Working Party Retrospective Data Mining Study. J Clin Oncol. 2015;33:3144‐3151. 26240227 10.1200/JCO.2014.59.1339

[26] Carré M , Porcher R , Finke J , et al. Role of age and hematopoietic cell transplantation‐specific comorbidity index in myelodysplastic patients undergoing an allotransplant: a retrospective study from the chronic malignancies working party of the European Group for Blood and Marrow Transplantation. Biol Blood Marrow Transplant. 2020;3:451‐457. 10.1016/j.bbmt.2019.10.015 31647984

[27] Oken MM , Creech RH , Tormey DC , et al. Toxicity and response criteria of the Eastern Cooperative Oncology Group. Am J Clin Oncol. 1982;5(6):649‐655. 7165009

[28] Koreth J , Schlenk R , Kopecky KJ , et al. Allogeneic stem cell transplantation for acute myeloid leukemia in first complete remission: systematic review and meta‐analysis of prospective clinical trials. JAMA. 2009;301(22):2349‐‐2361. 19509382 10.1001/jama.2009.813 PMC3163846

[29] Scott BL , Pasquini MC , Logan BR , et al. Myeloablative versus reduced‐intensity hematopoietic cell transplantation for acute myeloid leukemia and myelodysplastic syndromes. J Clin Oncol. 2017;35:1154‐1161. 28380315 10.1200/JCO.2016.70.7091 PMC5455603

[30] Hourigan CS , Dillon LW , Gui G , et al. Impact of conditioning intensity of allogeneic transplantation for acute myeloid leukemia with genomic evidence of residual disease. J Clin Oncol. 2020;38(12):1273‐1283. 31860405 10.1200/JCO.19.03011 PMC7164487

[31] Solomon SR , St. Martin A , Shah NN , et al. Myeloablative vs reduced intensity T‐cell‐replete haploidentical transplantation for hematologic malignancy. Blood Adv. 2019;3(19):2836‐2844. 31582392 10.1182/bloodadvances.2019000627 PMC6784523

[32] Spina F , Alessandrino PE , Milani R , et al. Allogeneic stem cell transplantation in therapy‐related acute myeloid leukemia and myelodysplastic syndromes: impact of patient characteristics and timing of transplant. Leuk Lymphoma. 2012;53:96‐102. 21740299 10.3109/10428194.2011.603445

[33] Spyridonidis A , Labopin M , Savani BN , et al. Redefining and measuring transplant conditioning intensity in current era: a study in acute myeloid leukemia patients. Bone Marrow Transplant. 2020;55(6):1114‐1125. 31996792 10.1038/s41409-020-0803-y

[34] Sorror ML , Storb RF , Sandmaier BM , et al. Comorbidity‐age index: a clinical measure of biologic age before allogeneic hematopoietic cell transplantation. J Clin Oncol. 2014;32:3249‐3256. 25154831 10.1200/JCO.2013.53.8157 PMC4178523

[35] Gratwohl A , Hermans J , Goldman JM , et al. Risk assessment for patients with chronic myeloid leukaemia before allogeneic blood or marrow transplantation. Chronic Leukemia Working Party of the European Group for Blood and Marrow Transplantation. Lancet. 1998;352:1087‐1092. 9798583 10.1016/s0140-6736(98)03030-x

[36] Gagelmann N , Bacigalupo A , Rambaldi A , et al. Haploidentical stem cell transplantation with posttransplant cyclophosphamide therapy vs other donor transplantations in adults with hematologic cancers: a systematic review and meta‐analysis. JAMA Oncol. 2019;5:1739‐1748. 10.1001/jamaoncol.2019.3541 PMC6802371 31621796

