# Supplementary material for: Allogeneic stem cell transplant in patients with acute myeloid leukemia and karnofsky performance status score less than or equal to 80%: A study from the acute leukemia working party of the European Society for Blood and Marrow Transplantation (EBMT)
Source: Cancer Med. 2020 Nov 26;10(1):23–33. doi: 10.1002/cam4.3593 (PMC7826477; doi:10.1002/cam4.3593)
Supplement: Supplementary file 1 — Supplementary Material [file CAM4-10-23-s001.docx]

**Supplementary material**

**Table S1. Conditioning regimens**

|  | **MAC** n=1244 (42%) | **RIC** n=1719 (58%) |
| --- | --- | --- |
| **Bu-Cy** | 319 ( 25.5% ) |  |
| **Bu-Flu** | 308 ( 24.6% ) | 714 ( 41.7% ) |
| **Cy-TBI** | 269 ( 21.5% ) |  |
| **Fluda-TBI** | 53 ( 4.2% ) | 248 ( 14.5% ) |
| **Fluda-Cy-TBI** |  | 119 ( 7% ) |
| **Flu-Mel** |  | 164 ( 9.6% ) |
| **TBF** | 93 ( 7.4% ) | 46 ( 2.7% ) |
| **Flu-Treo** | 41 ( 3.3% ) | 66 ( 3.9% ) |
| **Flamsa-TBI** |  | 42 ( 2.5% ) |
| **Flamsa-BU** |  | 93 ( 5.4% ) |
| **Other** | 168 (13.5%) | 218 (12.7)% |

Conditioning intensity was defined according to EBMT standards. Only protocols administered to 40 or more patients were reported. Abbreviations: Bu, Busulfan; Cy, Cyclophosphamide; FLAMSA, fludarabine, amsacrine, Ara-C, cyclophosphamide; Flu, Fludarabine; MAC, myeloablative conditioning; Mel, Melphalan; RIC, reduced-intensity conditioning; TBF, Thiotepa, Busulfan and Fludarabine; TBI, Total Body Irradiation; Treo, Treosulfan.

**Table S2. Multivariate analysis of transplant outcomes, stratified by KPS score, of patients receiving a MAC or RIC conditioning regimen**

**KPS = 80%**

| **Outcome** | | **HR (95% CI)** | ***P*** |
| --- | --- | --- | --- |
| **RI** | RIC vs MAC | 1.4 (1.13-1.71) | 0.002 |
|  | UD 10/10 vs MSD | 0.8 (0.631-0.94) | 0.01 |
| **NRM** | RIC vs MAC | 0.9 (0.663-1.1) | 0.2 |
|  | UD 10/10 vs MSD | 1.5 (1.15-1.94) | 0.002 |
| **LFS** | RIC vs MAC | 1.2 (0.986-1.36) | 0.07 |
|  | UD 10/10 vs MSD | 0.98 (0.841-1.15) | 0.8 |
| **OS** | RIC vs MAC | 1.1 (0.947-1.33) | 0.2 |
|  | UD 10/10 vs MSD | 1.03 (0.874-1.22) | 0.7 |
| **GRFS** | RIC vs MAC | 1.3 (1.12-1.49) | <0.001 |
|  | UD 10/10 vs MSD | 1.2 (1-1.33) | 0.05 |

**KPS < 80%**

| **Outcome** | | **HR (95% CI)** | ***P*** |
| --- | --- | --- | --- |
| **RI** | RIC vs MAC | 0.97 (0.584-1.61) | 0.9 |
|  | UD 10/10 vs MSD | 1.05 (0.657-1.68) | 0.8 |
| **NRM** | RIC vs MAC | 0.3 (0.179-0.517) | <0.001 |
|  | UD 10/10 vs MSD | 1.2 (0.717-2.16) | 0.4 |
| **LFS** | RIC vs MAC | 0.6 (0.402-0.826) | 0.003 |
|  | UD 10/10 vs MSD | 1.1 (0.796-1.62) | 0.5 |
| **OS** | RIC vs MAC | 0.5 (0.329-0.69) | <0.001 |
|  | UD 10/10 vs MSD | 1.1 (0.782-1.62) | 0.5 |
| **GRFS** | RIC vs MAC | 0.6 (0.45-0.885) | 0.008 |
|  | UD 10/10 vs MSD | 1.1 (0.797-1.53) | 0.6 |

Abbreviations: GRFS, graft-versus-host disease-free, relapse-free survival; KPS, Karnofsky performance status; LFS, leukemia free survival; MAC, myeloablative conditioning; MSD, matched sibling donor; NRM, non-relapse mortality; OS, overall survival; RI, relapse incidence; RIC, reduced-intensity conditioning; UD, unrelated donor.

**Table S3. Multivariate analysis of transplant outcomes on 1551 patients with available HCT-CI data**

| **Outcome** | | **HR (95% CI)** | ***P*** |
| --- | --- | --- | --- |
| **RI** | HCT-CI=0 (reference) | 1 |  |
|  | HCT-CI = 1 or 2 | 1.2 (0.917-1.57) | 0.19 |
|  | HCT-CI >2 | 1.24 (0.962-1.59) | 0.097 |
|  | KPS=80% vs <80% | 0.985 (0.74-1.31) | 0.92 |
| **NRM** | HCT-CI=0 (reference) | 1 |  |
|  | HCT-CI = 1 or 2 | 1.24 (0.894-1.71) | 0.20 |
|  | HCT-CI >2 | 1.39 (1.03-1.87) | 0.03 |
|  | KPS=80% vs <80% | 0.576 (0.429-0.774) | 0.0002 |
| **LFS** | HCT-CI=0 (reference) | 1 |  |
|  | HCT-CI = 1 or 2 | 1.22 (0.995-1.5) | 0.055 |
|  | HCT-CI >2 | 1.31 (1.08-1.59) | 0.0054 |
|  | KPS=80% vs <80% | 0.771 (0.629-0.945) | 0.012 |
| **OS** | HCT-CI=0 (reference) | 1 |  |
|  | HCT-CI = 1 or 2 | 1.27 (1.01-1.59) | 0.038 |
|  | HCT-CI >2 | 1.34 (1.09-1.66) | 0.0056 |
|  | KPS=80% vs <80% | 0.67 (0.541-0.83) | 0.00024 |
| **GRFS** | HCT-CI=0 (reference) | 1 |  |
|  | HCT-CI = 1 or 2 | 1.12 (0.93-1.35) | 0.23 |
|  | HCT-CI >2 | 1.17 (0.99-1.39) | 0.065 |
|  | KPS=80% vs <80% | 0.826 (0.685-0.995) | 0.045 |

Abbreviations: GRFS, graft-versus-host disease-free, relapse-free survival; HCT-CI, Hematopoietic Cell Transplantation-specific Comorbidity Index; KPS, Karnofsky performance status; LFS, leukemia free survival; NRM, non-relapse mortality; OS, overall survival; RI, relapse incidence;

**Table S4. List of centers contributing data to this study**

|  | N of patients |
| --- | --- |
| 267 Pessac [H Haut-Leveque] | 130 |
| 614 Hamburg [Univ H] | 95 |
| 207 Paris [St Louis] | 81 |
| 515 Helsinki [Univ Central H] | 78 |
| 246 Rotterdam [Erasmus MC] | 68 |
| 671 Lyon [H E Herriot] | 60 |
| 230 Marseille [Paoli Calmettes] | 59 |
| 546 Groningen [Univ H] | 52 |
| 717 Nottingham [City H] | 49 |
| 659 Brest [C.H.R.U Brest] | 48 |
| 661 Rennes [H Sud/Pontchaillou] | 48 |
| 727 Salamanca [H Clinico] | 47 |
| 666 Villejuif [Gustave Roussy] | 41 |
| 386 Bristol [Royal H Sick Chil] | 40 |
| 676 Vandoeuvre_Les_Nancy [H d`Enfants] | 40 |
| 565 Maastricht [Univ H] | 37 |
| 273 Clermont-Ferrand [Jean Perrin] | 35 |
| 556 Budapest [National Med Ctr] | 35 |
| 234 Brussels [St. Luc] | 34 |
| 731 Umeå [Univ H] | 34 |
| 277 Lille [H Claude Huriez] | 33 |
| 283 Lund [Univ H] | 33 |
| 672 Strasbourg [H Hautepierre] | 32 |
| 926 Montpellier [University] | 32 |
| 209 Leuven [Univ H] | 31 |
| 264 Poitiers [H La Miletrie] | 31 |
| 778 Sheffield [Royal Hallamshire] | 31 |
| 311 Wiesbaden [Kl Diagnostik] | 30 |
| 257 Dublin [St James] | 29 |
| 235 Oslo [Rikshospitalet] | 28 |
| 624 Toulouse [H Purpan] | 27 |
| 253 Nantes [Hotel Dieu] | 26 |
| 713 Leicester [Royal Infirmary] | 24 |
| 726 Liege [University] | 24 |
| 931 Suzhou [First Soochow] | 24 |
| 204 Ulm [Innere Med III] | 23 |
| 212 Stockholm [Univ H] | 23 |
| 977 Limoges [CHRU] | 23 |
| 994 Istanbul [Nightingale] | 23 |
| 203 Leiden [Univ H] | 22 |
| 202 Basel [202] | 21 |
| 513 Munich [Kl Grosshadern] | 21 |
| 808 Dresden [Universitaets Kl] | 21 |
| 919 Antalya [Medic Park H] | 21 |
| 206 Copenhagen [Rigshospitalet] | 20 |
| 304 Firenze [Careggi-Meyer] | 20 |
| 523 Nice [H de l`ARCHET I] | 20 |
| 237 Nijmegen [St Radboud] | 19 |
| 251 Caen [Hopital, Hematol] | 19 |
| 259 Essen [Univ H] | 19 |
| 656 Prague [Ist Hematology] | 19 |
| 295 Hannover [Medical Univ] | 18 |
| 389 Leipzig [Univ, Haemat/Oncol] | 18 |
| 718 Pilsen [Charles Univ H] | 18 |
| 307 Rome [Univ S Cuore] | 17 |
| 658 Bergamo [Ospedale, ematol] | 17 |
| 810 Freiburg [University] | 17 |
| 930 Moscow [NRC Haem.] | 17 |
| 239 Utrecht [University] | 16 |
| 270 Grenoble [H A Michallon] | 16 |
| 825 Alessandria [SS Ant e Bia] | 16 |
| 215 Brussels [Jules Bordet] | 15 |
| 289 Goeteborg [Sahlgrenska Univ H] | 15 |
| 680 Muenster [University] | 15 |
| 704 Southampton [General H] | 15 |
| 787 Regensburg [University] | 15 |
| 225 Turku [University] | 14 |
| 233 Besancon [H Jean Minjoz] | 14 |
| 250 Saint_Etienne [St Etienne] | 14 |
| 613 Barcelona [H Trias i Pujol] | 14 |
| 660 Reggio_Emilia [S Maria Nuova] | 14 |
| 705 Udine [Univ H] | 14 |
| 725 St._Petersburg [Pavlov Med Univ] | 14 |
| 244 Glasgow [Royal Infirmary] | 13 |
| 272 Tours [H Bretonneau] | 13 |
| 625 Nuernberg [Klinikum] | 13 |
| 729 Hradec_Králové [Charles U H, Hem] | 13 |
| 266 Uppsala [Univ H] | 12 |
| 606 Cuneo [S Croce e Carle] | 12 |
| 145 Stuttgart [Robert_Bosch_Kh] | 11 |
| 223 Tuebingen [Univ] | 11 |
| 262 Paris [Pitie-Salpetriere] | 11 |
| 501 Liverpool [Royal Univ H] | 11 |
| 552 Goettingen [Univ Kl] | 11 |
| 588 Amsterdam [VU Univ Med Ctr] | 11 |
| 746 Tartu [Univ H] | 11 |
| 807 Berlin [Charité Univ] | 11 |
| 268 Belfast [City H] | 10 |
| 589 Adana [Baskent Univ] | 10 |
| 996 Antwerp_Edegem [UZA] | 10 |
| 214 Barcelona [H Clinic] | 9 |
| 258 Jerusalem [Univ Hadassah] | 9 |
| 387 Birmingham [Queen Elizabeth] | 9 |
| 397 Riyadh [King Faisal] | 9 |
| 428 Gliwice [Sklodowska] | 9 |
| 756 Rome [Tor Vergata] | 9 |
| 785 Homburg [Univ Saarland] | 9 |
| 955 Amiens [H Sud] | 9 |
| 231 Torino [S. Giovanni (CTO)] | 8 |
| 252 Creteil [H Mondor Hematol] | 8 |
| 623 Verona [Policlinico] | 8 |
| 644 Vilnius [Santariskiy Kl] | 8 |
| 650 Angers [CHRU] | 8 |
| 813 Milano [S Raffaele] | 8 |
| 941 Rouen [Becquerel] | 8 |
| 169 Ankara [Gazi Univ] | 7 |
| 248 Pescara [Osp Civile] | 7 |
| 254 Leeds [St James] | 7 |
| 256 Kiel [UKSH] | 7 |
| 261 Geneva [261] | 7 |
| 276 Newcastle-Upon-Tyne [Freeman H] | 7 |
| 348 Aachen [RWTH] | 7 |
| 409 Petach-Tikva [Beilinson H] | 7 |
| 544 Monza [Osp S Gerardo] | 7 |
| 592 Idar-Oberstein [Kl Knochenmarktr] | 7 |
| 633 Teheran [Shariati] | 7 |
| 634 Aarhus [Univ, Hematol] | 7 |
| 663 Valencia [H Univ La Fe] | 7 |
| 744 Gent [Univ H] | 7 |
| 141 Brescia [Civili, Adulti] | 6 |
| 163 Piacenza [Osp Civile] | 6 |
| 228 Edinburgh [Western General] | 6 |
| 242 Santander [Valdecilla] | 6 |
| 297 Frankfurt am Main [Goethe-Univ] | 6 |
| 693 Warsaw [Inst Haematology] | 6 |
| 759 Barcelona [H Univ Bellvitge] | 6 |
| 208 Zürich [208] | 5 |
| 224 London [UCL] | 5 |
| 291 Porto [Inst Oncologia] | 5 |
| 354 Milano [Trap Mid Osseo] | 5 |
| 456 Pretoria [Albert Albert] | 5 |
| 524 Heidelberg [Medizinische Kl] | 5 |
| 530 Greifswald [Ernst-Moritz-Arndt] | 5 |
| 534 Cologne [Univ, Medicine] | 5 |
| 543 Modena [Policlinico] | 5 |
| 594 Linz [Elisabethinen H] | 5 |
| 617 Ankara [Ibni Sina H] | 5 |
| 646 Roeselare [AZ Delta] | 5 |
| 677 Katowice [Silesian Med Acad] | 5 |
| 692 Palermo [La Maddalena] | 5 |
| 740 Linköping [Univ H] | 5 |
| 754 Tel-Hashomer [Univ Adults] | 5 |
| 763 London [Kings College H] | 5 |
| 772 Cape_Town [Constantiaberg] | 5 |
| 775 Paris [St Antoine] | 5 |
| 809 Erlangen [University] | 5 |
| 205 London [Hammersmith] | 4 |
| 260 Barcelona [SCreu i S Pau] | 4 |
| 290 Karlsruhe [Klinikum] | 4 |
| 303 Cardiff [Univ Wales] | 4 |
| 558 Munich [Rechts der Isar] | 4 |
| 616 Milano [INT] | 4 |
| 640 Ljubljana [Univ Med Ctr] | 4 |
| 735 Murcia [H M Meseguer] | 4 |
| 780 Manchester [Christie] | 4 |
| 795 Pisa [Az Osp Univ] | 4 |
| 160 Paris [H Necker] | 3 |
| 218 London [Royal Marsden] | 3 |
| 232 Rome [Emat, La Sapienza] | 3 |
| 236 Madrid [Princesa] | 3 |
| 238 Córdoba [Reina Sofia] | 3 |
| 309 Madrid [Jiménez Díaz] | 3 |
| 339 Antwerp [AZ Stuivenberg] | 3 |
| 392 Palermo [Osp V Cervello] | 3 |
| 434 Singapore [Gen H] | 3 |
| 518 Berlin [HELIOS Kl] | 3 |
| 533 Jena [Friedrich-Schiller] | 3 |
| 561 Thessaloniki [Papanicolaou G H] | 3 |
| 597 Brno [Univ H] | 3 |
| 601 Manchester [Royal Infirmary] | 3 |
| 630 Brussels [Univ H] | 3 |
| 645 Marburg [Philipps Univ] | 3 |
| 749 Oldenburg [Klinikum] | 3 |
| 766 Napoli [Federico II] | 3 |
| 769 Sevilla [Virgen del Rocio] | 3 |
| 788 Ancona [Umberto I] | 3 |
| 792 Catania [Osp Ferrarotto] | 3 |
| 816 Warsaw [Military Med Acad] | 3 |
| 134 Bonn [Uni] | 2 |
| 161 Tel_Aviv [Sourasky] | 2 |
| 168 Ankara [Hacettepe Univ] | 2 |
| 211 Sao_Paulo [H Sirio-Libanes] | 2 |
| 305 Torino [Regina Margherita] | 2 |
| 359 Magdeburg [vGuericke U] | 2 |
| 367 Luebeck [Schleswig-Holstein] | 2 |
| 412 Ankara [Bayinder H] | 2 |
| 502 Venezia [SS Giovani e Paolo] | 2 |
| 538 Wroclaw [Ctr Cell Transpl] | 2 |
| 566 Cambridge [Addenbrookes H] | 2 |
| 577 Pamplona [H de Navarra] | 2 |
| 587 Reggio_Calabria [Centro Trapianti] | 2 |
| 602 Bremen [Kl Bremen-Mitte] | 2 |
| 642 Oviedo [H Covadonga] | 2 |
| 665 Clamart [H Percy] | 2 |
| 737 Pamplona [Cl Univ Navarra] | 2 |
| 798 Christchurch [Canterbury Health] | 2 |
| 806 Lyon [H Debrousse] | 2 |
| 104 Chemnitz [Kl Chemnitz] | 1 |
| 119 Ascoli_Piceno [Osp Mazzoni] | 1 |
| 142 Mannheim [Univ] | 1 |
| 146 Stuttgart [Diakonissen Kh] | 1 |
| 152 Augsburg [Zentral Kl] | 1 |
| 183 Tunis [Ctr Nat Gref Moel] | 1 |
| 217 Genova [S Martino] | 1 |
| 245 Parma [Centro Trapianti] | 1 |
| 282 Valencia [H Clinico] | 1 |
| 286 Pavia [S Matteo] | 1 |
| 294 Milano [Osp Niguarda] | 1 |
| 302 Zagreb [Univ H Rebro] | 1 |
| 308 Graz [Medical Univ] | 1 |
| 321 Siena [Le Scotte] | 1 |
| 323 Murcia [V Arrixaca] | 1 |
| 329 Porto [H S Joao] | 1 |
| 338 Halle [Univ Martin-Luther] | 1 |
| 374 Campinas [Zeferino Vaz] | 1 |
| 390 Duesseldorf [H Heine U] | 1 |
| 440 Kocaeli [Anadolu] | 1 |
| 444 Riyadh [Aziz] | 1 |
| 526 San_Giovanni_Rotondo [IRCCS] | 1 |
| 570 Santiago_De_Compostela [H Clin Univ] | 1 |
| 574 Olomouc [Univ H] | 1 |
| 598 San_Sebastian [H Aranzazu] | 1 |
| 610 Bratislava [Univ H] | 1 |
| 691 Ankara [Numune] | 1 |
| 712 Wuerzburg [Medizinische Kl II] | 1 |
| 768 London [S Bartholomew`s] | 1 |
| 797 Vicenza [Osp S Bartolo] | 1 |
| 811 Cagliari [Osp Businco] | 1 |
| 858 Jeddah [King Faisal] | 1 |
| 868 Lecce [Osp Vita Fazzi] | 1 |
| 925 St._Petersburg [FCHBE] | 1 |

**Appendix (list of contributors)**

University Hospital, Essen, Germany; Helsinki University Central Hospital, Helsinki, Finland; Universitätsklinikum Dresden, Dresden, Germany; University of Freiburg, Freiburg, Germany; Hopital St. Louis, Paris, France; Deutsche Klinik für Diagnostik, Wiesbaden, Germany; University Hospital Eppendorf, Hamburg, Germany; Hannover Medical University, Hannover, Germany; University Hospital Leipzig, Leipzig, Germany; CHU Bordeaux, Pessac, France; Silesian Medical Academy, Katowice, Poland; Centre For Clinical Haematology, Birmingham, United Kingdom; Erasmus MC-Daniel den Hoed Cancer Centre, Rotterdam, Netherlands, The; Hopital E. Herriot, Lyon, France; Nottingham City Hospital, Nottingham, United Kingdom; University Medical Centre, Utrecht, Netherlands, The; Centre Pierre et Marie Curie, Alger, Algeria; University Hospital, Basel, Switzerland; Sezione di Ematologia, Perugia, Italy; University Hospital Gasthuisberg, Leuven, Belgium; Medizinische Klinik und Poliklinik, Ulm, Germany; Huddinge University Hospital, Huddinge, Sweden; Hopital Claude Huriez, Lille, France; Charles University Hospital, Pilsen, Czech Republic; Hopital de Purpan, Toulouse, France; Hopital Saint Antoine, Paris, France; University Medical Center St. Radboud, Nijmegen, Netherlands, The; Charité Universitätsmedizin Berlin, Berlin, Germany; Tel-Aviv University, Tel-Hashomer, Israel; King Faisal Specialist Hospital & Research Centre, Riyadh, Saudi Arabia; Leiden University Hospital, Leiden, Netherlands, The; Hôpital Henri Mondor, Creteil, France; CHU Nantes, Nantes, France; St. James Hospital Trinity College, Dublin, Ireland; University of Heidelberg, Heidelberg, Germany; Service d`Onco Hematologie, Strasbourg, France; Ospedali Riuniti di Bergamo, Bergamo, Italy; Klinikum Nürnberg, Nürnberg, Germany; Universität Tübingen, Tübingen, Germany; Hopital Jean Minjoz, Besancon, France; Hôpital Necker, Paris, France; Unité de transplantation et de thérapie cellulaire, Marseille, France; Royal Marsden Hospital, London, United Kingdom; GKT School of Medicine, London, United Kingdom; Klinikum Grosshadern, Munich, Germany; Policlinico San Matteo, Pavia, Italy; George Papanicolaou General Hospital, Thessaloniki, Thessaloniki, Greece; University of Münster, Münster, Germany; Erciyes Medical School, Kayseri, Turkey; Cliniques Universitaires St. Luc, Brussels, Belgium; Bologna University, S.Orsola-Malpighi Hospital, Bologna, Italy; Hopital A. Michallon, Grenoble, France; Gazi Universitesi Tip Fakültesi, Ankara, Turkey; University College London Hospital, London, United Kingdom; Univ.`La Sapienza`, Rome, Italy; CHU Lapeyronie, Montpellier, France; Hospital Pitie-Salpetriere, Paris, France; Ospedale di Careggi, Firenze, Italy; Hopital La Miletrie, Poitiers, France; University Hospital, Uppsala, Sweden; Ankara University Faculty of Medicine, Ankara, Turkey; University Regensburg, Regensburg, Germany; University Hospital, Lund, Sweden; Centre Henri Becquerel, Rouen, France; Hospital U. Marqués de Valdecilla, Santander, Spain; VU University Medical Center, Amsterdam, Netherlands, The; Bristol Royal Hospital for Children, Bristol, United Kingdom; Royal Victoria Infirmary, Newcastle-Upon-Tyne, United Kingdom; University of Liege, Liege, Belgium; University of Saarland, Homburg, Germany; Istituto Scientifico H.S. Raffaele, Milano, Italy; Department of Haematology, Oxford, United Kingdom; Philipps Universitaet Marburg, Marburg, Germany; UnitT de Transplantation MTdullaire, Vandoeuvre Les Nancy, France; Rome Transplant Network, Rome, Italy; Rikshospitalet, Oslo, Norway; Royal Free Hospital and School of Medicine, London, United Kingdom; Glasgow Royal Infirmary, Glasgow, United Kingdom; Ospedale San Martino, Genova, Italy; Klinik für Knochenmarktransplantation, Idar-Oberstein, Germany; Hopitaux Universitaires de Geneve, Geneva, Switzerland; Johannes-Gutenberg-University, Mainz, Germany; Imperial College, London, United Kingdom; CHRU, Service des Maladies du Sang, Angers, France; Medizinische Universität Wien, Vienna, Austria; Haematology Department, Leeds, United Kingdom; Sahlgrenska University Hospital, Goeteborg, Sweden; Addenbrookes Hospital, Cambridge, United Kingdom; Southampton General Hospital, Southampton, United Kingdom; Bone Marrow Transplant Unit L 4043, Copenhagen, Denmark; Hospital Santa Creu i Sant Pau, Barcelona, Spain; University Hospital, Linköping, Sweden; Christie NHS Trust Hospital, Manchester, United Kingdom; Hospital Clínico, Salamanca, Spain; Dept. Haematology and Stem Cell Transplant, Budapest, Hungary; Institut Jules Bordet, Brussels, Belgium; Institute of Hematology and Blood Transfusion, Prague, Czech Republic; Centre Hospitalier Universitaire de Rennes, Rennes, France; Hadassah University Hospital, Jerusalem, Israel; Charité - Campus Benjamin Franklin, Berlin, Germany; Fédération de Greffe de Moelle et de, Clermont-Ferrand, France; Div. Stem Cell Transplantation and Immunotherapy, Kiel, Germany; Royal Liverpool University Hospital, Liverpool, United Kingdom; Ospedale San Gerardo, Monza, Italy; ICO – Hospital Duran i Reynals, Barcelona, Spain; North Trent BMT Programme (Adults), Sheffield, United Kingdom; Hôpital de l`ARCHET I, Nice, France; Vilnius University Hospital `Santariskiu Klinikos`, Vilnius, Lithuania; Manchester Royal Infirmary, Manchester, United Kingdom; S. Bortolo Hospital, Vicenza, Italy; University Hospital Maastricht, Maastricht, Netherlands, The; Leicester Royal Infirmary, Leicester, United Kingdom; Medical University of Gdansk, Gdansk, Poland; University Hospital, Zürich, Switzerland; Azienda Ospedaliera S. Giovanni, Torino, Italy; Umea University Hospital, Umeå, Sweden; Ospedale di Niguarda Ca` Granda, Milano, Italy; University Hospital, Udine, Italy; Medical University Graz, Graz, Austria; Hôpital Percy, Clamart, France; St. Bartholomew`s and The Royal London Hospital, London, United Kingdom; Turku University, Turku, Finland; Ospedale V. Cervello, Palermo, Italy; A.Z. Sint-Jan, Brugge, Belgium; Universita degli Studi di Bari, Bari, Italy; Ospedale La Maddalena - Dpt. Oncologico, Palermo, Italy; Ospedale Ferrarotto, Catania, Italy; University Medical Center Groningen (UMCG), Groningen, Netherlands, The; Tel Aviv Sourasky Medical Center, Tel Aviv, Israel; Ospedale Civile, Pescara, Italy; Ospedale Maggiore di Milano, Milano, Italy; Birmingham Heartlands Hospital, Birmingham, United Kingdom; RP Group Royal Perth Hospital, Perth, Australia; University of Napoli, Napoli, Italy; Universitätsklinikum Göttingen, Gottingen, Germany; Hospital Universitari Germans Trias i Pujol, Barcelona, Spain; University Hospital Erlangen, Erlangen, Germany; Hopital Nord, Saint Etienne Cedex 2, France; Elisabethinen-Hospital, Linz, Austria; University of Wales, Cardiff, United Kingdom; Institut Gustave Roussy, Villejuif, France; Hospital Clinic, Barcelona, Spain; University of Cologne, Cologne, Germany; Hacettepe University, Ankara, Turkey; University Hospital, Olomouc, Czech Republic; Hospital Universitario La Fe, Valencia, Spain; University Hospital Brno, Brno, Czech Republic; Evangelismos Hospital, Athens, Greece; Constantiaberg Medi-Clinic, Cape Town, South Africa; Central Clinical Hospital, Warsaw, Poland; AK St. Georg., Hamburg, Germany; Heinrich Heine Universität, Düsseldorf, Germany; King Hussein Cancer Centre, Amman, Jordan; Centre Hospitalier Universitaire, Caen, France; Hospital Vall d`Hebron, Barcelona, Spain; Ege University Medical School, Bornova-Izmir, Turkey; University Med. Center, Ljubljana, Slovenia; CHU Morvan, Brest, France; Institute of Haematology and Blood Transfusion, Warsaw, Poland; Charles University Hospital, Hradec Králové, Czech Republic; Academisch Ziekenhuis bij de Universiteit, Amsterdam, Netherlands, The; Belfast City Hospital, Belfast, United Kingdom; Inst. Portugues de Oncologia do Porto, Porto, Portugal; Universita Cattolica S. Cuore, Rome, Italy; K. Marcinkowski University of Medical Science, Poznan, Poland; Rambam Medical Center, Haifa, Israel; Policlinico G.B. Rossi, Verona, Italy; SPb State I. Pavlov Medical University, St. Petersburg, Russia; H SS. Antonio e Biagio, Alessandria, Italy; Klinikum Augsburg, Augsburg, Germany; DCTK, Wroclaw, Poland; Canterbury Health Laboratories, Christchurch, New Zealand; Hosp. Reina Sofia, Córdoba, Spain; Friedrich-Schiller-Universität Jena, Jena, Germany; Azienda Ospedaliera, Reggio Calabria, Italy; Hospital Gregorio Marañón, Madrid, Spain; Patras University Medical School, Patras, Greece; Univ. Est. de Campinas/TMO/UNICAMP, Campinas, Brazil; University Hospital VUB, Brussels, Belgium; Hospital Clínico Universitario, Valencia, Spain; Antwerp University Hospital (UZA), Antwerp Edegem, Belgium; Azienda Ospedali Riuniti di Ancona, Ancona-Torrette, Italy; University Hospital Gent, Gent, Belgium; Plymouth Hospitals NHS Trust, Plymouth, United Kingdom; Inst. Portugues Oncologia, Lisboa, Portugal; Hospital Covadonga, Oviedo, Spain; Robert-Bosch-Krankenhaus, Stuttgart, Germany; Ernst-Moritz-Arndt-Universität Greifswald, Greifswald, Germany; Klinikum Rechts der Isar, Munich, Germany; Hospital Morales Meseguer, Murcia, Spain; Hospital `Virgen del Rocio`, Sevilla, Spain; Western General Hospital, Edinburgh, United Kingdom; ZNA, Antwerp, Belgium; Beilinson Hospital, Petach-Tikva, Israel; Hospital Aranzazu, San Sebastian, Spain; Dipartimento di Oncologia, dei trapianti e delle, Pisa, Italy; Ospedale S. Camillo-Forlanini, Rome, Italy; Hospital San Maurizio, Bolzano, Italy; Allogeneic Stem Cell Transplant Center, Würzburg, Germany; Baskent University Hospital, Adana, Turkey; University Hospital, Bratislava, Slovakia; Tartu University Hospital, Tartu, Estonia; St. George`s Hospital, London, United Kingdom; Guy`s Hospital, London, United Kingdom; Karadeniz Technical University, Trabzon, Turkey; Policlinico Le Scotte, Siena, Italy; Hospital de Gran Canaria `Dr Negrin`, Las Palmas De Gran Canaria, Spain; AZ. Spedali Civili- Brescia Universitu of Brescia, Brescia, Italy; Hospital de la Princesa, Madrid, Spain; Specialized Children`s Oncohematpology Hospital, Sofia, Bulgaria; Hospital del SAS, Cádiz, Spain; Haukeland University Hospital, Bergen, Norway; University Hospital Innsbruck, Innsbruck, Austria; Hospedale Nord, Taranto, Italy; Ankara Numune Education and, Ankara, Turkey; Medical University of Lublin, Lublin, Poland; Ospedale A. Businco, Cagliari, Italy; IRCCS, Casa Sollievo della Sofferenza, San Giovanni Rotondo, Italy; Az. Ospedaliera S. Croce e Carle, Cuneo, Italy; Clinica Puerta de Hierro, Madrid, Spain; Kliniken Essen Süd, Essen, Germany; NHS Grampian, Aberdeen, United Kingdom; Fundeni Clinical Institute, Bucharest, Romania; Uni. Modena, Policlinico, Modena, Italy; KLINIKUM BREMEN - MITTE, Bremen, Germany; Mazzoni Hospital, Ascoli Piceno, Italy; Martin-Luther-Universität Halle-Wittenberg, Halle, Germany; Pesaro Hospital, Pesaro, Italy; Istanbul Tip Fakueltesi, Istanbul, Turkey; Wroclaw Medical University, Wroclaw, Poland; Medical Park Hospitals, Antalya, Turkey; Military Medical Academy, Belgrade, Serbia and Montenegro; University of Milano, Milano, Italy; Shariati Hospital, Teheran, Iran; A.O.R.N. `SAN.G MOSCATI`, Avellino, Italy; Unità Operativa Oncoematologia Pediatrica, Pisa, Italy; P.O. `R. Binaghi`, Cagliari, Italy; Military Medical Academy, Warsaw, Poland; University of Medicine and Pharmacy, Timisoara, Romania; GATA BMT Center, Ankara, Turkey; Universität Rostock, Rostock, Germany; Osmangazi University, Fac. of Medicine, Eskisehir, Turkey; Sectia Clinica de Hematolgoie si, Targu-Mures, Romania; The Trustee of London Clinic, London, United Kingdom; Istituto Clinico Humanitas, Milano, Italy; Ankara Oncology Research & Education Hospital, Ankara, Turkey; Hospital Universitario Son Espases, Palma De Mallorca, Spain; Cerrahpasa Medical School, Istanbul, Turkey; Hospital Univ. Virgen de las Nieves, Granada, Spain; National Haematology Centre, Riga, Latvia; Hospital Ampang, Ampang, Malaysia; Klinikum Oldenburg, Oldenburg, Germany; Campus Charité Mitte, Berlin, Germany; Univ. of Torino, Torino, Italy; American University of Beirut, Beirut, Lebanon; Ankara Bayindir Hospital, Haematology BMT, Ankara, Turkey; Hospital de Navarra, Pamplona, Spain; Wellington Hospital, Wellington, New Zealand; Belorussian Centre for Paediatric, Minsk, Belarus; Heilig Hartziekenhuis, Roeselare, Belgium; Hospital C. Panico, Tricase (Lecce), Italy; Arcispedale S. Maria Nuova, Reggio Emilia, Italy; Medical Academy, Wroclaw, Poland; CHRU Limoges, Limoges, France; Clinic Frankfurt (Oder) GmbH of Internal Medicine, Frankfurt (Oder), Germany; Hospital Universitario Virgen de la Arrixaca, Murcia, Spain; Ospedale Civile SS. Giovanni e Paolo, Venezia, Italy; Alfred Hospital, BMT Programme, Melbourne, Australia; Schneider Children`s Medical Center of Israel, Petach-Tikva, Israel; Institute G. Gaslini, Genova, Italy; Ospedale San Gerardo, Monza, Italy; Klinikum Karlsruhe gGmbH, Karlsruhe, Germany; IRCCS Policlinico San Matteo, Pavia, Italy; Hospital Clinico Universitario, Santiago De Compostela, Spain; Hospital de Santa Maria, Lisboa, Portugal; St.Savas Oncology Hospital, Athens, Greece; Univ. di Palermo, Palermo, Italy; Azienda Ospedaliera Universitaria San Martino, Genova, Italy; Klinikum der Johann-Wolfgang Goethe Universität, Frankfurt am Main, Germany; C.H.U. Timone Enfants, Marseille, France; Univ. of Parma, Parma, Italy; Clinica di Oncoematologia Pediatrica, Padova, Italy; University Hospital Center Rebro, Zagreb, Croatia; CHNDRF, Charleroi, Belgium; University Hospital Motol, Prague, Czech Republic; University of Cape Town Faculty of Health Sciences, Cape Town, South Africa; Istituto per l`Infanzia `Burlo Garofolo`, Trieste, Italy; University Hospital, Tübingen, Germany; Jagiellonian University, Krakow, Poland; University Children`s Hospital, Graz, Austria; Cardarelli Hospital, Napoli, Italy; Hospital Ramon y Cajal, Madrid, Spain; Clínica Universitaria de Navarra, Pamplona, Spain; IHOP, Lyon, France; Ospedale San Carlo, Potenza, Italy; Kreiskrankenhaus Hameln, Hameln, Germany; Hospital Guglielmo da Saliceto, Piacenza, Italy; Albert Aberts Stem Cell Transplant Unit, Pretoria, South Africa; Adnan Menderes University Med. Faculty, Aydin, Turkey; Spedali Civili - Brescia, Brescia, Italy; European Institute of Oncology, Milano, Italy; Hospital Juan Canalejo, La Coruña, Spain; ITMO-Instituto de Transplante de Medula Osea, La Plata, Argentina; St. Anna Kinderspital, Vienna, Austria; Chaim Sheba Medical Center, Tel-Hashomer, Israel; Hospital Carlos Haya, Málaga, Spain; Pediatric University Teaching Hospital, Bratislava, Slovakia; Dokuz Eylül Universitesi, Izmir, Turkey; Royal Hospital for Sick Children, Glasgow, United Kingdom; Musgrove Park Hospital (Somerset), Taunton, United Kingdom; The Children`s Hospital at Westmead, Sydney, Australia; Marmara University, Istanbul, Turkey; Niño Jesus Children`s Hospital, Madrid, Spain; University of Jena, Jena, Germany; University Hospital, Collegium Medicum UMK, Bydgoszcz, Poland; Royal Liverpool Children`s NHS Trust, Liverpool, United Kingdom; Our Lady`s Hospital for Sick Children, Dublin, Ireland; University of Bologna, Bologna, Italy; Wilhelminenspital, Vienna, Austria; Pédiatrie et Génétique Médicale, Rouen, France.
